# Effects of Simultaneous Application of Double Chelating Agents to Pb-Contaminated Soil on the Phytoremediation Efficiency of *Indocalamus decorus* Q. H. Dai and the Soil Environment

**DOI:** 10.3390/toxics10120713

**Published:** 2022-11-22

**Authors:** Yixiong Yang, Mingyan Jiang, Jiarong Liao, Zhenghua Luo, Yedan Gao, Weiqian Yu, Rui He, Shihan Feng

**Affiliations:** College of Landscape Architecture, Sichuan Agricultural University, Chengdu 611130, China

**Keywords:** EDTA, GLDA, *Indocalamus decorus* Q. H. Dai, NTA, Pb availability, rhizosphere

## Abstract

Recent studies have shown that the combined application of ethylenediaminetetraacetic acid (EDTA) and degradable chelating agents can enhance EDTA’s affinity for heavy metals and reduce its toxicity, but the effect of this combination on the phytoremediation remains largely unknown. This study evaluated and compared the effects of EDTA, nitrilotriacetic acid (NTA), and glutamic acid-N,N-diacetic acid (GLDA) alone (E, N, G treatment), and in combination (EN and EG treatment), on the growth of dwarf bamboo (*Indocalamus decorus* Q. H. Dai), their phytoremediation efficiency, and the soil environment in Pb-contaminated soil. The results showed that treatment E significantly reduced the biomass, while treatments N and EN were more conducive to the distribution of aerial plant biomass. Except for treatment E, the total Pb accumulation in all treatments increased significantly, with the highest increase in treatment EN. For double chelating agents, the acid-soluble Pb concentrations in rhizosphere and non-rhizosphere soils of treatments EN and EG were lower than those of treatment E, and the soil water-soluble Pb content after 20 days of treatment EN was significantly lower than that of treatment EG. Furthermore, chelating agents generally increased soil-enzyme activity in rhizosphere soil, indicating that chelating agents may promote plant heavy-metal uptake by changing the rhizosphere environment. In conclusion, treatment EN had the highest phytoremediation efficiency and significantly lower environmental risk than treatments E and EG, highlighting its massive potential for application in phytoremediation of Pb-contaminated soil when combined with *I. decorus*.

## 1. Introduction

Accumulation of heavy metals in soil poses a serious threat to the entire biosphere [1]. It can be caused by various human activities, including mining, pesticides and fertilizers, industrial production, irrigation using wastewater, and electronic-waste disposal [2,3]. Lead (Pb) is one of the most widely distributed heavy metals in soil [4]. It is not biodegradable and adversely affects the microorganisms and plants in the soil environment. It accumulates in different parts of the plant and enters the food chain, causing serious harm to human health [5]. Phytoremediation has many merits, such as low cost and green environmental protection, compared to the traditional soil remediation technologies [6,7]. Currently, hyperaccumulators are considered the ideal plant species for phytoremediation. However, these species are scarce, with most of them characterized by slow growth and low biomass [8]. In contrast, some non-hyperaccumulators, such as bamboo, are fast-growing species with higher total biomass but with lower heavy-metal accumulation capacity than hyperaccumulators [9]. Studies postulate that some bamboo species have good tolerance and Pb-accumulation ability under Pb stress [10]. Our previous study showed dwarf bamboo, such as *Sasa fortunei* (Van Houtte) Fiori, *Sasa agrenteostriata* (Regel) E. G. Gamus, and *Indocalamus decorus* Q. H. Dai have strong tolerance, a reasonable detoxification mechanism, and high lead-accumulation ability under Pb stress [11,12,13].

In recent years, chelating agents have been widely used to assist phytoremediation by improving the availability of heavy metals in the soil and enhancing the accumulation capacity of plants [14,15,16]. Ethylenediaminetetraacetic acid (EDTA) is one of the most effective chelating agents for enhancing Pb accumulation in plants [17,18]. However, EDTA remains in the soil for a long time, causing toxicity, thus leading to secondary pollution [19,20]. Some studies suggest that the combined application of EDTA and degradable chelating agents to assist phytoremediation enhances EDTA’s affinity for heavy metals and reduces its toxicity [21]. In recent years, nitrilotriacetic acid (NTA) has been widely used in phytoremediation experiments because of its strong chelating ability and ease of biodegradation which improve the ability of plants to extract heavy metals from the soil [22,23,24]. A new biodegradable chelating agent, glutamic acid-N,N-diacetic acid (GLDA), has also gradually attracted the attention of researchers. It has a good chelating ability with various metal ions and excellent biodegradability, with a significantly smaller ecological footprint than traditional chelating agents. GLDA has been used to wash soils contaminated with heavy metals as a phytoremediation strategy [25,26,27]. However, the effect of the combined application of EDTA and NTA/GLDA on the phytoremediation of lead-contaminated soils remains largely unknown.

This study evaluated and compared the effects of EDTA, NTA, and GLDA, alone and in combination, on the growth of dwarf bamboo (*I. decorus*), their phytoremediation efficiency, and soil environment in Pb-contaminated soil.

## 2. Materials and Methods

### 2.1. Soil Preparation and Experimental Materials

The experiment was conducted in pots in Sichuan Agricultural University (Chengdu, Sichuan Province, Southwest China). The soils were topsoil (0–20 cm) collected from a field near the school. The soil was air-dried and then passed through a 5 mm sieve before use. The properties of the soil are as follows: pH of 6.81, organic matter of 2.64%, available nitrogen of 100.08 mg/kg, available phosphorus of 87.74 mg/kg, available potassium of 151.45 mg/kg, and total Pb of 28.45 mg/kg. The soil was adequately mixed with (CH_3_COOH)_2_Pb·3H_2_O (Sigma-Aldrich Co., Ltd., St. Louis, MI, USA) to obtain contaminated soil with a Pb concentration of 1500 mg/kg (1500 mg/kg was determined based on our previous study [11]) before the experimental setup.

*I. decorus* seedlings were sourced from a nursery near the university. EDTA and NTA were purchased from Sigma-Aldrich Co., Ltd. GLDA was purchased from Akzo Nobel Chemicals Co., Ltd. (Singapore).

### 2.2. Experimental Setup

The experiment was set up in July 2019. *I. decorus* seedlings were transplanted into pots 16.5 cm in diameter and 26.5 cm high with holes in the bottom. The pots were held in plastic trays and contained 3 kg (dry weight) of Pb-contaminated soil. Thirty seedlings were planted in each pot, with every treatment replicated thrice. The soils attached to the root-rhizome systems were carefully removed before transplanting to avoid damaging the root, and the plants were subsequently pruned to obtain consistent biomass among replicates. The field water-holding capacity of the soil was maintained at 75% using deionized water. Chelating agents were added to each treatment after 40 days, as outlined in Table 1. The chelating agents used in each treatment were dissolved in 200 mL of deionized water and then slowly and evenly poured into the potted soil. The control experiment was treated with an equal volume of deionized water.

Soil samples were collected on days 45, 50, 55, 60, 65, and 70 to determine the water-soluble Pb content in the soil. All bamboo plants were harvested after 70 days, and the rhizosphere soil (soil about 1 mm thick attached to the roots [28]) was subsequently collected. The harvested plants were separated into roots, stems, leaves, and rhizomes and then soaked in 20 mM EDTA-2Na solution for 15 min to remove the attached metal ions on the surface. All plant organs were subsequently washed thoroughly with deionized water and processed for analysis. Soil samples from each pot were also collected, mixed, air-dried, and sieved for analysis.

### 2.3. Plant Analysis

#### 2.3.1. Plant Biomass

The harvested bamboo organs were oven-dried at 105 °C for 15 min, followed by drying at 80 °C until a constant weight was attained, to determine the biomass of each organ. The tissue samples were then ground, sieved through a 0.15 mm mesh, and stored in air-tight plastic bags before analysis of the Pb level.

#### 2.3.2. Pb Level in Plant Organs

The Pb concentration in the plant organs was determined using a method described by Cai et al. (2021) [12]. Plant samples (0.2 g) were digested with HNO_3_ and HClO_4_ (*v*:*v*, 5:1), followed by determination of the Pb concentrations using an atomic absorption spectrophotometer (Shimadzu AA-7000, Kyoto, Japan). Pb accumulation in the bamboo was calculated by multiplying the Pb concentration of each organ with its biomass.

#### 2.3.3. BCF and TF Analysis

The biological concentration factor (BCF) and translocation factor (TF) were calculated according to the following formulae [29]:

BCF = C_plant_/C_soil_

TF = C_aerial part_/C_underground part_

where C_plant_ (mg/kg) is the Pb concentration in the plant and C_soil_ (mg/kg) is 1500 (initial Pb concentration in soil).

### 2.4. Soil Analysis

#### 2.4.1. Water-Soluble Pb

The water-soluble Pb content in the soil was determined using a method described by Wang et al. (2009) [30]. The soil was first mixed with deionized water in a ratio of 2.5:1, followed by mixing of the mixture for 30 min through shaking. The mixture was then centrifuged and filtered through a 0.45 μm filter membrane to obtain the filtrate. The filtrate was acidified with HNO_3_, and its Pb content was subsequently analyzed using an atomic absorption spectrophotometer.

#### 2.4.2. Morphological Pb Analysis

Pb distribution forms in the soil were determined using the Community Bureau of Reference (BCR) sequential extraction method [31]. A half gram of soil from each treatment was used for continuous extraction experiments. Table S1 outlines the specific steps and chemical fractions of Pb. The supernatant was collected after each extraction step through centrifugation and subsequent filtration, followed by its acidification using HNO_3_ determination of its Pb level using an atomic absorption spectrophotometer.

#### 2.4.3. Determination of Soil-Enzyme Activities

Soil urease, invertase, and catalase activities were determined using the sodium phenate–sodium hypochlorite colorimetric method [32], the 3,5-dinitrosalicylic acid colorimetric method [33], and the enzyme-linked colorimetric assay [34], respectively.

### 2.5. Statistical Analysis

Statistical analyses were performed using the SPSS 20.0 software and expressed as means of three replicates. Differences between treatments were determined using one-way ANOVA at a significance threshold of *p* < 0.05. The resultant data were then analyzed with Excel 2010 and graphed.

## 3. Results

### 3.1. Plant Biomass

**Figure 1:**
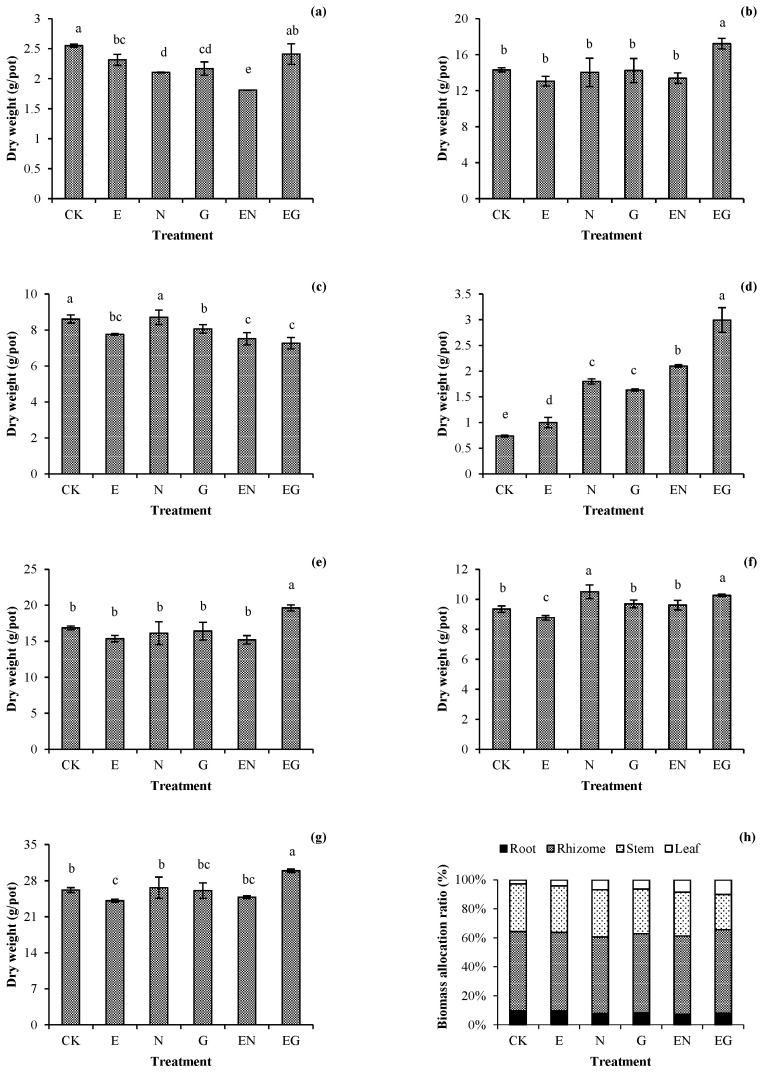
Effects of the different treatments on the (**a**) root, (**b**) rhizome, (**c**) stem, (**d**) leaf, (**e**) underground part, (**f**) aerial part, (**g**) total dry weight, and (**h**) biomass allocation ratio of dwarf bamboo. Error bars represent the standard deviations (SDs) of the means (*n* = 3). According to Duncan’s multiple range test, different letters above the bars indicate significant differences (*p* < 0.05).

Figure 1 presents the effects of different treatments on the biomass of *I. decorus*. The biomass of the underground plant organs in each treatment was not significantly different from that of CK. However, the biomass of the underground organs in the EG treatment was significantly higher than that of CK. Notably, the E treatment was the only treatment with significantly lower aerial-part biomass than CK. Moreover, it was the only treatment whose total plant biomass decreased significantly (0.92 times that of CK). The growth of plants in other treatments was not inhibited. N and EN treatments were the most conducive in the distribution of biomass of the aerial plant parts, with an increase of 3.78% and 3.08%, respectively, compared to that in the CK treatment.

### 3.2. Pb Level in Plants

**Figure 2:**
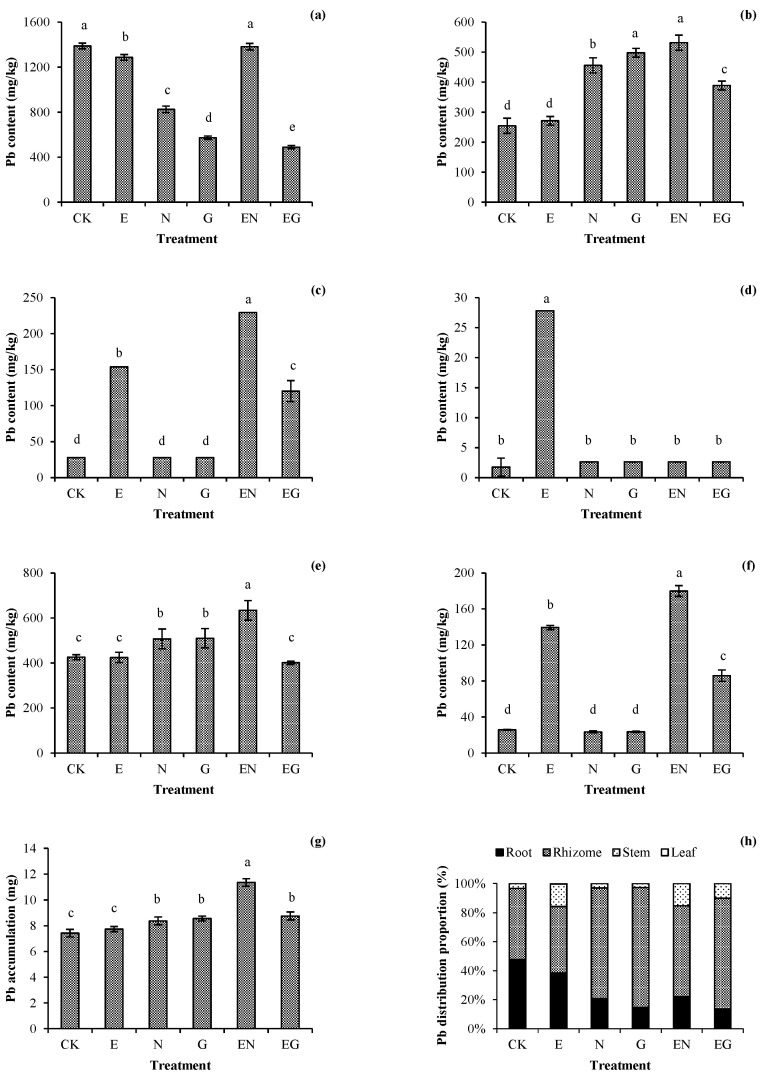
Effects of the different treatments on the (**a**) root, (**b**) rhizome, (**c**) stem, (**d**) leaf, (**e**) aerial-part, and (**f**) underground-part Pb concentrations; (**g**) total Pb accumulation; and (**h**) Pb distribution patterns in different tissues of dwarf bamboo. Error bars represent the standard deviations (SDs) of the means (*n* = 3). According to Duncan’s multiple range test, different letters above the bars indicate significant differences (*p* < 0.05).

Figure 2 shows the effect of different treatments on the Pb level in plants. There was no significant increase in the Pb content in the plant roots in all treatments as compared with CK (Figure 2a). The Pb content of the rhizomes in all treatments increased significantly except for plants in the E treatment, with the EN treatment having the highest increase (2.09 times that of CK, Figure 2b). Plants in the N and G treatments did not have any further increase in the Pb content in the aerial plant parts. The Pb content in their stems and leaves was not significantly different from those of CK. Plants in the E, EN, and EG treatments had a significant increase in the Pb content in the stems, with those in the EN treatment having the highest increase (8.24 times that of CK, Figure 2c). Of note, only the E treatment significantly increased the Pb content in plant leaves (Figure 2d).

The underground parts of plants in the N, G, and EN treatments had a significant increase in the Pb content, with those in the EN treatment having the highest increase (1.53 times that of CK, Figure 2e). The aerial parts of plants in the E, EN, and EG treatments had a significant increase in the Pb content, with those in the EN treatment having the highest increase (6.98 times that of CK, Figure 2f). Generally, the total Pb accumulation of plants in all treatments increased compared to CK, with EN having the maximum increase (1.53 times that of CK, Figure 2g). The distribution ratio of Pb in plants suggested that all treatments could reduce the proportion of Pb accumulation in roots and promote the transfer of Pb to rhizomes or stems. However, the proportion of Pb accumulation in the plant leaves in all treatments was very low, indicating that Pb transportation into the leaves of *I. decorus* was limited even with the assistance of chelating agents (Figure 2h).

### 3.3. Bioaccumulation and Transfer of Pb in Plants

Table 2 shows the BCF and TF values of *I. decorus* under different treatments. The BCF value of the underground parts in each treatment did not significantly decrease compared to that of CK, with the EN treatment having the maximum value (1.5 times that of CK). In contrast, there was a significant increase in the BCF value of the aerial parts in E, EN, and EG treatments compared to CK, with the EN treatment having a maximum value (6.0 times that of CK). These results suggested that the EN treatment caused a plant-growth and lead-absorption balance. Its TF value was lower than that of the E treatment (84.85% of E treatment) but significantly higher than that of other treatments.

### 3.4. Water-Soluble Pb in Soil

**Figure 3:**
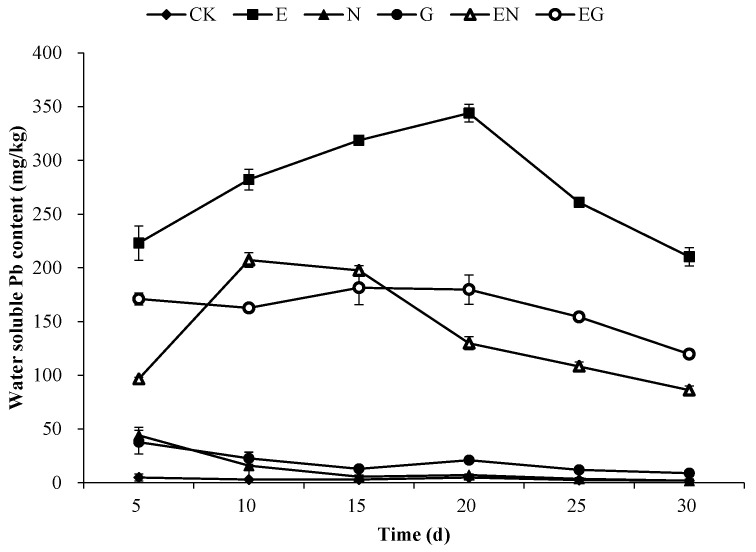
Changes in the soil water-soluble Pb content over 30 days.

Figure 3 shows the changes in the water-soluble Pb content over the 30 days of applying the chelating agents in the different treatments. The water-soluble Pb content in the N and G treatments exhibited a decreasing trend with time, while the E, EN, and EG treatments exhibited an increase first and then a decrease. Notably, the water-soluble Pb content in the E treatment was higher than in other treatments over the 30 days (46.41–117.89 times that of CK). The water-soluble Pb content in EN treatment after 20 days was lower than that of E and EG treatments.

### 3.5. Morphological Distribution of Pb in the Soil

**Figure 4:**
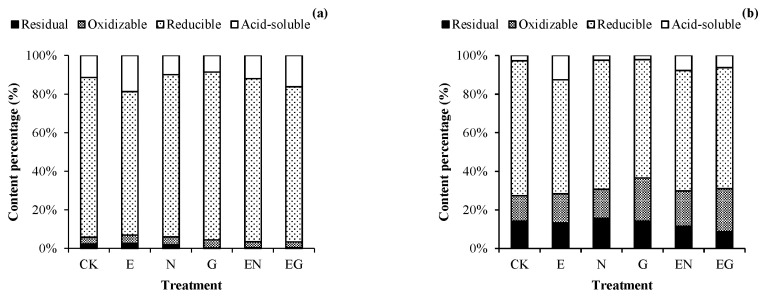
Effects of different treatments on the morphological distribution of Pb in (**a**) rhizosphere soil and (**b**) non-rhizosphere soil.

Figure 4 shows the morphological distribution of Pb in the rhizosphere and non-rhizosphere soils under different treatments. The proportion of acid-soluble Pb in the rhizosphere and non-rhizosphere soils in all treatments increased except in the N and G treatments. The E treatment had the highest increase, with an increase of 7.34% (rhizosphere) and 9.91% (non-rhizosphere) compared to the proportion of acid-soluble Pb in CK. Of note, the proportion of acid-soluble Pb and reducible Pb in the rhizosphere soil of each treatment was higher than that of non-rhizosphere soil (1.48–4.21 times and 1.19–1.42 times that of non-rhizosphere soil, respectively). In contrast, the oxidizable Pb and residual Pb proportion in the rhizosphere soil of each treatment were lower than that of the non-rhizosphere soil (0.14–0.29 times and 0.02–0.20 times that of non-rhizosphere soil, respectively).

### 3.6. Soil-Enzyme Activity

Table 3 outlines the effects of different treatments on soil-enzyme activities of plant rhizosphere and non-rhizosphere soils. The enzyme activity of rhizosphere soil in all treatments was higher than that of non-rhizosphere soil (1.07–4.52 times that of non-rhizosphere soil). However, each treatment had different soil-enzyme-activity changes in the two soil zones. Generally, the chelating agents increased soil-enzyme activity in rhizosphere soil but inhibited these activities in non-rhizosphere soil. This phenomenon was attributed to the positive effect of combining the chelating agent and root exudates, which changed the soil activity. In contrast, the non-rhizosphere zone had no root system, and thus the chelating agent had a negative effect in changing the soil activity.

## 4. Discussion

### 4.1. Effects of Applying Chelating Agents on Plant Growth

Adding chelating agents enhances Pb availability in the soil, thus negatively affecting plant growth [21,30]. In this study, plant growth in the E treatment was significantly inhibited, a finding consistent with those of Han et al. (2018) [35]. This phenomenon is attributed to the high affinity of EDTA for heavy metals, which has a negative impact on the mineral balance in the soil, leading to cell-metabolism disorder and destabilization of biofilm [36]. Plant growth in the remaining treatments was not inhibited, possibly because of the fast degradation rate of NTA and GLDA [37]. Their impact on the soil environment was limited because of their short half-life. NH_3_ is their major by-product and is required for plant growth [38]. Interestingly, combining the two chelating agents exhibited contrasting results. Plants in the EN treatment had the lowest biomass among all treatments except those in the E treatment, possibly because its high Pb accumulation resulted in a stronger Pb toxic effect. In contrast, plants in the EG treatment had the highest biomass among all treatments, attributed to the low Pb content in the roots, which exerted minimal toxic effects on the plants, thus promoting growth.

### 4.2. Effects of Chelating Agents on Pb Uptake in Plants and Phytoremediation Efficiency

Pb absorbed by plants accumulates mainly in roots and is difficult to transfer to the aerial parts of plants [39,40,41,42]. Studies postulate that chelating agents increase the translocation of Pb from the underground to the aerial parts of plants [43,44,45,46]. The reason for this effect is still unclear and varies depending on the types of chelating agents, plants, and heavy metals [47]. In this study, the Pb content in the roots of plants in each treatment decreased because of the addition of chelating agents, which promoted the transportation of Pb from the roots to other plant parts. Interestingly, different chelating agents had different effects on Pb transport in *I. decorus*. E treatment significantly increased Pb transport to the aerial plant parts (stems and leaves), while N and G treatments only increased Pb transport to the rhizomes. However, the application of NTA or GLDA in combination with EDTA increased Pb transport to the aerial parts of the plants. This phenomenon was attributed to the Pb–chelator complex reducing the binding of Pb to the extracellular cation exchange sites, thereby increasing the Pb flux through the apoplast [48,49]. The destruction of the Casparian band by EDTA may have also led to an increase in the transport of Pb to the aerial part of the plant [50].

Phytoremediation efficiency depends on the plant’s biomass production and the concentration of heavy metals in the plant. Accumulation of heavy metals in plants is thus more important than their concentration in plants [51]. Studies postulate that applying EDTA, NTA, or GLDA increases the accumulation of heavy metals in plants [11,38,52]. In this study, total Pb accumulation in plants treated with chelating agents was significantly higher than in those in the CK treatment. However, there was no significant difference between Pb accumulation in plants in E and CK treatments, possibly because of the decreased biomass in plants in the E treatment. The maximum Pb accumulation in plants was observed in the EN treatment. These findings were consistent with those of Wang et al. (2019) [38]. This study suggested that combining chelating agents favored the accumulation of heavy metals in plants more than a single chelating agent because of the additive effect between two chelating agents. The combined application also makes the concentration of the two chelating agents reach a more suitable level. Cui et al. (2007) demonstrated that Pb accumulation in *Zinnia elegans* seedlings decreased with the addition of EDTA in a concentration-dependent manner [53].

### 4.3. Effect of Chelating Agents on the Soil Environment

Chelating agents mobilize the metal ions by enhancing their desorption from the soil, thereby promoting their uptake by plants [54]. In this study, the water-soluble Pb content in the soil increased significantly in all treatments after applying the chelating agent. This phenomenon is attributed to the combining of the chelating agent with the free Pb ions in the soil solution to form complexes that release the Pb bound to the soil components/cation exchange sites and reduce the adsorption/precipitation of Pb from the soil solution. Many factors affect the Pb chelation process. The metal-chelating agent’s stability constant is the most important factor in this process [55]. The stability constant of Pb chelation with EDTA is higher than that of GLDA and NTA [56]. The water-soluble and acid-soluble Pb content in the soil under E treatment was thus higher than in other treatments. Moreover, the content of the above two forms of Pb in the soil under E treatment remained at a very high level after 30 days because of the non-biodegradability of EDTA, thereby posing a high environmental risk. Notably, the water-soluble and acid-soluble Pb content in the soil treated with biodegradable chelator alone had dropped to an extremely low level at 30 days, findings consistent with the previously reported degradation time of NTA and GLDA [37]. The water-soluble Pb content in the soils treated with combined chelating agents was lower than that of soil under E treatment. This finding suggests that the combined application of the chelating agents can reduce the environmental risks caused by EDTA.

Herein, the acid-soluble Pb content of non-rhizosphere soil was lower than that of rhizosphere soil in the same treatment. In contrast, the content of oxidizable Pb and residual Pb in non-rhizosphere soil was higher than that of rhizosphere soil. This phenomenon was attributed to the plant roots changing the rhizosphere environment by secreting organic acids, thus improving the mobility of Pb in the environment [57]. Studies postulate that EDTA can release lead from soil particles, especially from iron-manganese oxides and organic matter [58]. This assertion is consistent with our findings that the proportion of reducible Pb in the two soils was lower after E, EN, and EG treatment than in CK.

Changes in the soil-enzyme activity are among the most direct responses to the soil micro-ecological environment [59]. Some studies indicate that the enzyme activity of rhizosphere soil is 1.3–2 times that of non-rhizosphere soil and decreases as the distance from the root increases [60]. These reports are consistent with the results of this study in which the soil-enzyme activities were higher in the rhizosphere than in the non-rhizosphere under the same treatment. Furthermore, in this study, chelating agents generally increased soil-enzyme activity in rhizosphere soil, indicating that chelating agents may promote plant heavy-metal uptake by changing the rhizosphere environment. Nonetheless, there were certain differences in the activity changes of different soil enzymes, possibly dependent on the Pb availability in the soil and the nature of the chelating agent. The specific reasons for these differences should thus be further studied.

## 5. Conclusions

This study focused on evaluating the effects of chelating agents on *I. decorus* growth, phytoremediation efficiency, and the soil environment in Pb-contaminated soil. All the chelating agents except E did not inhibit plant growth, with N and EN treatments being the most conducive in enhancing biomass increase in the aerial plant parts. Moreover, the application of chelating agents effectively improved Pb accumulation and transport capacity of plants, especially the EN treatment. There was a greater decrease in the water-soluble and acid-soluble Pb content in the soils under EN and EG treatment than in soil under E treatment. Notably, the water-soluble Pb content in the soil under EN treatment was lower than that under EG treatment after 20 days, indicating that the environmental risk of EN treatment was lower than that of E and EG treatment. Furthermore, chelating agents generally increased soil-enzyme activity in rhizosphere soil, indicating that chelating agents may promote plant heavy-metal uptake by changing the rhizosphere environment. EN treatment combined with planting *I. decorus* is the most promising strategy for phytoremediation of Pb-contaminated soil. However, the reproducibility of these results and their utility for practical application should be verified using field trials.

## Figures and Tables

**Table 1:** Soil treatment with chelating agents.

Treatment	Soil Pb Concentration (mg/kg)	Type and Concentration of Chelating Agent (mg/kg)
CK	1500	-
E	1500	EDTA 1500
N	1500	NTA 1500
G	1500	GLDA 1500
EN	1500	EDTA 750 + NTA 750
EG	1500	EDTA 750 + GLDA 750

**Table 2:** Effects of different treatments on BCF and TF values of dwarf bamboo. All data are shown as the means ± SDs (*n* = 3). Different lowercase letters indicate that the mean values are significantly different among the treatments according to Duncan’s multiple range test (*p* < 0.05).

Treatment	BCF		TF
	Underground Part	Aerial Part	
CK	0.28 ± 0.01 c	0.02 ± 0.00 d	0.06 ± 0.00 d
E	0.28 ± 0.01 c	0.09 ± 0.00 b	0.33 ± 0.01 a
N	0.34 ± 0.01 b	0.02 ± 0.00 d	0.05 ± 0.00 d
G	0.34 ± 0.01 b	0.02 ± 0.00 d	0.05 ± 0.00 d
EN	0.42 ± 0.01 a	0.12 ± 0.00 a	0.28 ± 0.01 b
EG	0.27 ± 0.01 c	0.06 ± 0.01 c	0.21 ± 0.02 c

**Table 3:** Effects of different treatments on the enzyme activity of rhizosphere and non-rhizosphere soil. All data are shown as the means ± SDs (*n* = 3). Different lowercase letters indicate that the mean values are significantly different among the treatments according to Duncan’s multiple range test (*p* < 0.05).

Treatment	Soil-Enzyme Species (Rhizosphere)			Soil-Enzyme Species (Non-Rhizosphere)		
	Urease (mg/g)	Catalase (mg/g)	Invertase (mg/g)	Urease (mg/g)	Catalase (mg/g)	Invertase (mg/g)
CK	0.67 ± 0.05 b	5.15 ± 0.27 c	45.61 ± 0.94 ab	0.25 ± 0.01 ab	4.52 ± 0.12 a	39.01 ± 1.38 b
E	0.85 ± 0.04 a	4.14 ± 0.12 d	42.15 ± 0.89 bc	0.26 ± 0.01 a	1.65 ± 0.25 d	33.21 ± 1.26 d
N	0.87 ± 0.04 a	6.17 ± 0.32 b	44.60 ± 2.89 ab	0.19 ± 0.01 c	4.79 ± 0.33 a	36.91 ± 2.01 bc
G	0.71 ± 0.07 b	5.78 ± 0.19 b	46.62 ± 2.03 a	0.18 ± 0.02 cd	4.86 ± 0.22 a	43.47 ± 1.28 a
EN	0.86 ± 0.03 a	5.85 ± 0.12 b	39.61 ± 1.44 c	0.23 ± 0.01 b	3.94 ± 0.25 b	35.31 ± 0.41 cd
EG	0.67 ± 0.03 b	7.64 ± 0.23 a	42.11 ± 3.80 bc	0.17 ± 0.01 d	3.51 ± 0.06 c	36.24 ± 2.54 bc

## Data Availability

The authors declare that [the/all other] data supporting the findings of this study are available within the article.

## Supplementary Materials

The following supporting information can be downloaded at: https://www.mdpi.com/article/10.3390/toxics10120713/s1, Table S1: Chemical reagents and analytical conditions for the modified BCR sequential extraction method.

## References

[1] Elturk M., Abdullah R., Rozainah M., Abu Bakar N.K. (2018). Evaluation of heavy metals and environmental risk assessment in the Mangrove Forest of Kuala Selangor estuary, Malaysia. Mar. Pollut. Bull..

[2] Jiang B., Adebayo A., Jia J., Xing Y., Deng S., Guo L., Liang Y., Zhang D. (2018). Impacts of heavy metals and soil properties at a Nigerian e-waste site on soil microbial community. J. Hazard. Mater..

[3] Wang P., Li Z., Liu J., Bi X., Ning Y., Yang S., Yang X. (2019). Apportionment of sources of heavy metals to agricultural soils using isotope fingerprints and multivariate statistical analyses. Environ. Pollut..

[4] Datko-Williams L., Wilkie A., Richmond-Bryant J. (2013). Analysis of U.S. soil lead (Pb) studies from 1970 to 2012. Sci. Total Environ..

[5] Kushwaha A., Hans N., Kumar S., Rani R. (2018). A critical review on speciation, mobilization and toxicity of lead in soil-microbe-plant system and bioremediation strategies. Ecotoxicol. Environ. Saf..

[6] Cao X., Wang X., Tong W., Gurajala H.K., Lu M., Hamid Y., Feng Y., He Z., Yang X. (2019). Distribution, availability and translocation of heavy metals in soil-oilseed rape (*Brassica napus* L.) system related to soil properties. Environ. Pollut..

[7] Gabriele I., Race M., Papirio S., Esposito G. (2021). Phytoremediation of pyrene-contaminated soils: A critical review of the key factors affecting the fate of pyrene. J. Environ. Manag..

[8] Mahar A., Wang P., Ali A., Awasthi M.K., Lahori A.H., Wang Q., Li R., Zhang Z. (2016). Challenges and opportunities in the phytoremediation of heavy metals contaminated soils: A review. Ecotoxicol. Environ. Saf..

[9] Bian F., Zhong Z., Zhang X., Yang C., Gai X. (2019). Bamboo–An untapped plant resource for the phytoremediation of heavy metal contaminated soils. Chemosphere.

[10] Zhong B., Chen J., Shafi M., Guo J., Wang Y., Wu J., Ye Z., He L., Liu D. (2017). Effect of lead (Pb) on antioxidation system and accumulation ability of Moso bamboo (*Phyllostachys pubescens*). Ecotoxicol. Environ. Saf..

[11] Jiang M., Liu S., Li Y., Li X., Luo Z., Song H., Chen Q. (2019). EDTA-facilitated toxic tolerance, absorption and translocation and phytoremediation of lead by dwarf bamboos. Ecotoxicol. Environ. Saf..

[12] Cai X., Jiang M., Liao J., Yang Y., Li N., Cheng Q., Li X., Song H., Luo Z., Liu S. (2020). Biomass allocation strategies and Pb-enrichment characteristics of six dwarf bamboos under soil Pb stress. Ecotoxicol. Environ. Saf..

[13] Cai X., Liao J., Yang Y., Li N., Xu M., Jiang M., Chen Q., Li X., Liu S., Luo Z. (2021). Physiological resistance of Sasa argenteostriata (Regel) E.G. Camus in response to high-concentration soil Pb stress. Acta Physiol. Plant..

[14] Zhang X., Zhong B., Shafi M., Guo J., Liu C., Guo H., Peng D., Wang Y., Liu D. (2018). Effect of EDTA and citric acid on absorption of heavy metals and growth of Moso bamboo. Environ. Sci. Pollut. Res..

[15] Gul I., Manzoor M., Kallerhoff J., Arshad M. (2020). Enhanced phytoremediation of lead by soil applied organic and inorganic amendments: Pb phytoavailability, accumulation and metal recovery. Chemosphere.

[16] Gul I., Manzoor M., Hashmi I., Bhatti M.F., Kallerhoff J., Arshad M. (2019). Plant uptake and leaching potential upon application of amendments in soils spiked with heavy metals (Cd and Pb). J. Environ. Manag..

[17] Li H., Wang Q., Cui Y., Dong Y., Christie P. (2005). Slow release chelate enhancement of lead phytoextraction by corn (*Zea mays* L.) from contaminated soil—A preliminary study. Sci. Total Environ..

[18] Zhang H., Guo Q., Yang J., Ma J., Chen G., Chen T., Zhu G., Wang J., Zhang G., Wang X. (2016). Comparison of chelates for enhancing *Ricinus communis* L. phytoremediation of Cd and Pb contaminated soil. Ecotoxicol. Environ. Saf..

[19] He S., Wu Q., He Z. (2014). Synergetic effects of DA-6/GA 3 with EDTA on plant growth, extraction and detoxification of Cd by Lolium perenne. Chemosphere.

[20] Attinti R., Barrett K.R., Datta R., Sarkar D. (2017). Ethylenediaminedisuccinic acid (EDDS) enhances phytoextraction of lead by vetiver grass from contaminated residential soils in a panel study in the field. Environ. Pollut..

[21] Guo D., Ali A., Ren C., Du J., Li R., Lahori A.H., Xiao R., Zhang Z., Zhang Z. (2018). EDTA and organic acids assisted phytoextraction of Cd and Zn from a smelter contaminated soil by potherb mustard (*Brassica juncea*, Coss) and evaluation of its bioindicators. Ecotoxicol. Environ. Saf..

[22] Hseu Z.-Y., Jien S.-H., Wang S.-H., Deng H.-W. (2013). Using EDDS and NTA for enhanced phytoextraction of Cd by water spinach. J. Environ. Manag..

[23] Zhao L., Li T., Yu H., Zhang X., Zheng Z. (2016). Effects of [S,S]-ethylenediaminedisuccinic acid and nitrilotriacetic acid on the efficiency of Pb phytostabilization by Athyrium wardii (Hook.) grown in Pb-contaminated soils. J. Environ. Manag..

[24] Hu X., Liu X., Zhang X., Cao L., Chen J., Yu H. (2017). Increased accumulation of Pb and Cd from contaminated soil with Scirpus triqueter by the combined application of NTA and APG. Chemosphere.

[25] Wu Q., Cui Y., Li Q., Sun J. (2015). Effective removal of heavy metals from industrial sludge with the aid of a biodegradable chelating ligand GLDA. J. Hazard. Mater..

[26] Guo X., Zhao G., Zhang G., He Q., Wei Z., Zheng W., Qian T., Wu Q. (2018). Effect of mixed chelators of EDTA, GLDA, and citric acid on bioavailability of residual heavy metals in soils and soil properties. Chemosphere.

[27] Wang G., Zhang S., Zhong Q., Peijnenburg W.J., Vijver M.G. (2018). Feasibility of Chinese cabbage (*Brassica bara*) and lettuce (*Lactuca sativa*) cultivation in heavily metals−contaminated soil after washing with biodegradable chelators. J. Clean. Prod..

[28] Edwards J., Johnson C., Santos-Medellín C., Lurie E., Podishetty N.K., Bhatnagar S., Eisen J.A., Sundaresan V. (2015). Structure, variation, and assembly of the root-associated microbiomes of rice. Proc. Natl. Acad. Sci. USA.

[29] Anning A.K., Akoto R. (2018). Assisted phytoremediation of heavy metal contaminated soil from a mined site with Typha latifolia and Chrysopogon zizanioides. Ecotoxicol. Environ. Saf..

[30] Wang X., Wang Y., Mahmood Q., Islam E., Jin X., Li T., Yang X., Liu D. (2009). The effect of EDDS addition on the phytoextraction efficiency from Pb contaminated soil by Sedum alfredii Hance. J. Hazard. Mater..

[31] Rauret G., López-Sánchez J.F., Sahuquillo A., Rubio R., Davidson C., Ure A., Quevauviller P. (1999). Improvement of the BCR three step sequential extraction procedure prior to the certification of new sediment and soil reference materials. J. Environ. Monit..

[32] Willis R.B., Montgomery M.E., Allen P.R. (1996). Improved Method for Manual, Colorimetric Determination of Total Kjeldahl Nitrogen Using Salicylate. J. Agric. Food Chem..

[33] Gonçalves C., Rodriguez-Jasso R.M., Gomes N., Teixeira J.A., Belo I. (2010). Adaptation of dinitrosalicylic acid method to microtiter plates. Anal. Methods.

[34] Shang Z., Wu Z., Li D., Zhu P., Gao H., Zhang L., Gong P. (2012). The activity and kinetic parameters of oxidoreductases in phaeozem in response to long-term fertiliser management. J. Soil Sci. Plant Nutr..

[35] Han Y., Zhang L., Gu J., Zhao J., Fu J. (2018). Citric acid and EDTA on the growth, photosynthetic properties and heavy metal accumulation of Iris halophila Pall. cultivated in Pb mine tailings. Int. Biodeterior. Biodegradation.

[36] Ruley A.T., Sharma N.C., Sahi S.V., Singh S.R., Sajwan K.S. (2006). Effects of lead and chelators on growth, photosynthetic activity and Pb uptake in Sesbania drummondii grown in soil. Environ. Pollut..

[37] Borowiec M., Huculak M., Hoffmann K., Hoffmann J. (2009). Biodegradation of selected substances used in liquid fertilizers as an element of Life Cycle Assessment. Pol. J. Chem. Technol..

[38] Wang K., Liu Y., Song Z., Wang D., Qiu W. (2019). Chelator complexes enhanced Amaranthus hypochondriacus L. phytoremediation efficiency in Cd-contaminated soils. Chemosphere.

[39] Brunet J., Varrault G., Zuily-Fodil Y., Repellin A. (2009). Accumulation of lead in the roots of grass pea (*Lathyrus sativus* L.) plants triggers systemic variation in gene expression in the shoots. Chemosphere.

[40] Gupta D., Nicoloso F., Schetinger M., Rossato L., Pereira L., Castro G., Srivastava S., Tripathi R. (2009). Antioxidant defense mechanism in hydroponically grown Zea mays seedlings under moderate lead stress. J. Hazard. Mater..

[41] Jiang W., Liu D. (2010). Pb-induced cellular defense system in the root meristematic cells of *Allium sativum* L.. BMC Plant Biol..

[42] Gonçalves A.C., Schwantes D., de Sousa R.F.B., da Silva T.R.B., Guimarães V.F., Campagnolo M.A., de Vasconcelos E.S., Zimmermann J. (2020). Phytoremediation capacity, growth and physiological responses of Crambe abyssinica Hochst on soil contaminated with Cd and Pb. J. Environ. Manag..

[43] Neugschwandtner R.W., Tlustoš P., Komárek M., Száková J. (2008). Phytoextraction of Pb and Cd from a contaminated agricultural soil using different EDTA application regimes: Laboratory versus field scale measures of efficiency. Geoderma.

[44] Liu D., Islam E., Li T., Yang X., Jin X., Mahmood Q. (2008). Comparison of synthetic chelators and low molecular weight organic acids in enhancing phytoextraction of heavy metals by two ecotypes of Sedum alfredii Hance. J. Hazard. Mater..

[45] Barrutia O., Garbisu C., Hernández-Allica J., García-Plazaola J.I., Becerril J.M. (2010). Differences in EDTA-assisted metal phytoextraction between metallicolous and non-metallicolous accessions of *Rumex acetosa* L.. Environ. Pollut..

[46] Yan L., Li C., Zhang J., Moodley O., Liu S., Lan C., Gao Q., Zhang W. (2017). Enhanced Phytoextraction of Lead from Artificially Contaminated Soil by Mirabilis jalapa with Chelating Agents. Bull. Environ. Contam. Toxicol..

[47] Saifullah, Meers E., Qadir M., de Caritat P., Tack F., Du Laing G., Zia M. (2009). EDTA-assisted Pb phytoextraction. Chemosphere.

[48] Tandy S., Schulin R., Nowack B. (2006). The influence of EDDS on the uptake of heavy metals in hydroponically grown sunflowers. Chemosphere.

[49] Hernández-Allica J., Garbisu C., Barrutia O., Becerril J.M. (2007). EDTA-induced heavy metal accumulation and phytotoxicity in cardoon plants. Environ. Exp. Bot..

[50] Bell P.F., Mclaughlin M.J., Cozens G., Stevens D.P., Owens G., South H. (2003). Plant Uptake of 14 C-EDTA, 14 C-Citrate, and 14 C-Histidine from Chelator-Buffered and Conventional Hydroponic Solutions. Plant Soil.

[51] Zhan J., Zhang Q., Li T., Yu H., Zhang X., Huang H. (2019). Effects of NTA on Pb phytostabilization efficiency of Athyrium wardii (Hook.) grown in a Pb-contaminated soil. J. Soils Sediments.

[52] Yu H., Zhan J., Zhang Q., Huang H., Zhang X., Wang Y., Li T. (2020). NTA-enhanced Pb remediation efficiency by the phytostabilizer Athyrium wardii (Hook.) and associated Pb leaching risk. Chemosphere.

[53] Cui S., Zhou Q.-X., Wei S.-H., Zhang W., Cao L., Ren L.-P. (2007). Effects of exogenous chelators on phytoavailability and toxicity of Pb in Zinnia elegans Jacq. J. Hazard. Mater..

[54] Evangelou M.W., Ebel M., Schaeffer A. (2007). Chelate assisted phytoextraction of heavy metals from soil. Effect, mechanism, toxicity, and fate of chelating agents. Chemosphere.

[55] Saifullah, Shahid M., Zia-Ur-Rehman M., Sabir M., Ahmad H.R. (2015). Phytoremediation of Pb-Contaminated Soils Using Synthetic Chelates. Soil Remediation and Plants: Prospects and Challenges.

[56] Begum Z.A., Rahman I.M.M., Tate Y., Egawa Y., Maki T., Hasegawa H. (2012). Formation and Stability of Binary Complexes of Divalent Ecotoxic Ions (Ni, Cu, Zn, Cd, Pb) with Biodegradable Aminopolycarboxylate Chelants (dl-2-(2-Carboxymethyl)Nitrilotriacetic Acid, GLDA, and 3-Hydroxy-2,2′-Iminodisuccinic Acid, HIDS) in Aqueous Solutions. J. Solut. Chem..

[57] Schwab A., Zhu D., Banks M. (2008). Influence of organic acids on the transport of heavy metals in soil. Chemosphere.

[58] Udovic M., Lestan D. (2009). Pb, Zn and Cd mobility, availability and fractionation in aged soil remediated by EDTA leaching. Chemosphere.

[59] Duan C., Fang L., Yang C., Chen W., Cui Y., Li S. (2018). Reveal the response of enzyme activities to heavy metals through in situ zymography. Ecotoxicol. Environ. Saf..

[60] Kuzyakov Y., Razavi B.S. (2019). Rhizosphere size and shape: Temporal dynamics and spatial stationarity. Soil Biol. Biochem..

