# Supplementary material for: Effects of Simultaneous Application of Double Chelating Agents to Pb-Contaminated Soil on the Phytoremediation Efficiency of Indocalamus decorus Q. H. Dai and the Soil Environment
Source: Toxics. 2022 Nov 22;10(12):713. doi: 10.3390/toxics10120713 (PMC9781716; doi:10.3390/toxics10120713)
Supplement: Supplementary file 1 [file toxics-10-00713-s001.zip › toxics-2021109-supplementary.pdf]

Table S1. Chemical reagents and analytical conditions for the modified BCR sequential extraction method.

Step	Fraction	Operation
1	Acid-soluble state	20 mL of HOAc (0.11 M, pH 2.8) was added and shaken for 16 h to obtain the supernatant.
2	Reducible state	20 mL $NH_2OH \cdot HCl$ (0.5 M, pH 2) was added and shaken for 16 h to obtain the supernatant.
3	Oxidizable state	10 mL H_2O_2 was added to the samples and the samples were heated at 85 ℃ for 1 h; 5 mL H_2O_2 (30%, pH 2) was added again and the samples were heated at 85 ℃ for 1 h, then NH_4OAc (1 M, pH 2) was added and shaken for 16 h to obtain the supernatant.
4	Residual state	Digestion with HNO_3-$HClO_4$-H_2O_2.
